# A Chemical Analysis of Sodalite, a New Mineral from Greenland

**Published:** 1811-10

**Authors:** Thomas Thomson

**Affiliations:** Fellow of the Imperial Chirurgo-Medical Academy of Petersburgh


### *A Chemical Analysis of Sodalite, a new Mineral from Greenland.* *By* Thomas Thomson, M. D. F. R. S. E., Fellow of the Imperial Chirurgo-Medical Academy of Petersburgh.

(Trans. of Roy. Soc. Ed.)

THE mineral, to which I have given the name of *Sodalite*, was put into my hands by Mr. Allan. In the Greenland collection which he purchased, there were several specimens of a rock, obviously primitive. In the composition of these the substance of which I am about to treat formed a constituent, and, at first appearance, was taken for felspar, to which it bears a very striking resemblance.

This rock is composed of no less than five different fossils, namely, garnet, hornblende, augite, and two others, which form the paste of the mass. These are evidently different minerals; but in some specimens are so intimately blended, that it required the skill of Count Bournon to make the discrimination, and ascertain their real nature. Even this distinguished mineralogist was at first deceived by the external aspect, and considered the paste as common lamellated felspar, of a greenish colour. But a peculiarity, which presented itself to Mr. Allan in one of the minerals, induced him to call the attention of Count Bournon more particularly to its construction.

On a closer examination of the mineral, Mr. de Bournon found, that some small fragments, which he had detached, presented rectangular prisms, terminated by planes, measuring, with the sides of the prism, 110° and 70° or nearly so,—a form which belongs to a rare mineral, known by the name of sahlite from Sweden. He further observed, intermixed along with this, another mineral; and after some trouble, succeeded in detaching a mass, presenting a regular rhomboidal dodecahedron. It was to this form that Mr. Allan had previously requested his attention.

Some time before this investigation, Mr. de Bournon had examined a mineral from Sweden, of a lamellated structure, and a greenish colour, which, he found, indicated the same form. From this circumstance, together with some external resemblance which struck him, he was induced to conclude, that our mineral was a variety of that substance.

To that substance the name of Swedish *natrolite* had been given, in consequence of the investigation of Dr. Wollaston, who found that it contained a large proportion of soda.

There are few minerals, however, that are so totally distinct in their external characters as the natrolite of Klaproth, and and the substance we are now treating of. The mineral examined by Klaproth occurs at Roegan*, on the Lake of Constance, in porphyry-slate, coating the sides of veins and cavities in a mamellated form, the texture of which is compact, fibrous, and radiated; the colour pale yellow, in some places passing into white, and marked with brown zones. Hitherto it had never been found in a state sufficiently perfect to afford any indications of form. Lately, however, Mr. de Bournon was so fortunate as to procure some of it, presenting very delicate needleform crystals, which, by means of a strong magnifier, he was able to ascertain presented flat rectangular prisms, terminated by planes, which, he thought, might form angles of 60° and 120° with the sides of the prism. With this neither our mineral nor the Swedish can have any connection, farther than some analogy which may exist in their composition.

Concerning the Swedish mineral I have not been able to obtain much satisfactory information. There is a specimen of it in Mr. Allan's cabinet, which he received directly from Sweden, sent by a gentleman who had just before been in London, and was well acquainted with the collections of that city, from which it is inferred, that the specimen in question is the same as that examined by Count Bournon and Dr. Wollaston.

Werner has lately admitted into his system a new mineral species, which he distinguishes by the name of Fettstein. Of this I have seen two descriptions; one by Hauy, in his Tableau Comparatif, published last year; and another by Count Dunin Borkowski, published in the 69th volume of the Journal de Physique, and translated in Nicholson's Journal, (Vol. XXVI, p. 384.) The specimen, called Swedish natrolite, in Mr. Allan's possession, agrees with these descriptions in every particular, excepting that its specific gravity is a little higher. Borkowski states the specific gravity of fettstein at 2·563; Hauy at 2·6138; while I found the specific gravity of Mr. Allan's specimen to be 2·779, and, when in small fragments, to be as high as 2·790. This very near agreement in the properties of the Swedish natrolite with the characters of the fettstein leads me to suppose it the substance, to which Werner has given that name. This opinion is strengthened, by a fact mentioned by Haüy, that fettstein had been at first considered as a variety of wernerite. For the specimen sent to Mr. Allan, under the name of compact

* It has been observed also by Professor Jameson, in the floetz-trap rocks behind Burntisland.

wernerite, wernerite, is obviously the very same with the supposed natrolite of Sweden. Now, if this identity be admitted, it will follow, that our mineral constitutes a species apart. It bears, indeed, a considerable resemblance to it; but neither the crystalline form, nor the constituents of fettstein, as stated by Haüy, are similar to those of the mineral to which I have given the name of sodalite. The constituents of fettstein, as ascertained by Vauquelin, are as follows:

| | |
|---|---|
| Silica . . . . . | 44·00 |
| Alumina . . . . . | 34·00 |
| Oxide of iron . . . | 4·00 |
| Lime . . . . . | 0·12 |
| Potash and soda . . | 16·50 |
| Loss . . . . . | 1·38 |
| | 100·00 |

Sodalite, as has been already mentioned, occurs in a primitive rock, mixed with sahlite, augite*, hornblende, and garnet†.

It occurs massive; and crystallised, in rhomboidal dodecahedrons, which, in some cases, are lengthened, forming six-sided prisms, terminated by trihedral pyramids.

Its colour is intermediate between celandine and mountain green, varying in intensity in different specimens. In some cases it seems intimately mixed with particles of sahlite, which doubtless modify the colour.

External lustre glimmering, internal shining, in one direction vitreous, in another resinous.

Fracture foliated, with at least a double cleavage; cross fracture conchoidal.

Fragments indeterminate; usually sharp-edged.

Translucent.

Hardness equal to that of felspar. Iron scratches it with difficulty.

Brittle.

Easily frangible.

Specific gravity, at the temperature of 60°, 2·378. The specimen was not absolutely free from sahlite.

When heated to redness, does not decrepitate, nor fall to powder, but becomes dark gray, and assumes very nearly the appearance of the Swedish natrolite of Mr. Allan, which I

* The situation of the augite deserves attention. Hitherto it has been, with a few exceptions, found only in flœtz-trap rocks.

† The particular colour and appearance of the garnet shows, that the rock came from Greenland: for similar garnet has never been observed, except in specimens from Greenland.

consider as fettstein. If any particles of sahlite be mixed with it, they become very conspicuous, by acquiring a white colour, and the opacity and appearance of chalk. The loss of weight was 2·1 per cent. I was not able to melt it before the blow-pipe.

1. A hundred grains of the mineral, reduced to a fine powder, were mixed with 200 grains of pure soda, and exposed for an hour to a strong red heat, in a platinum crucible. The mixture melted, and assumed, when cold, a beautiful grass-green colour. When softened with water, the portion adhering to the sides of the crucible acquired a fine brownish-yellow. Nitric acid being poured upon it, a complete solution was obtained.

2. Suspecting, from the appearance which the fused mass assumed, that it might contain chromium, I neutralized the solution, as nearly as possible, with ammonia, and then poured into it a recently prepared nitrate of mercury. A white precipitate fell, which being dried, and exposed to a heat rather under redness, was all dissipated, except a small portion of gray matter, not weighing quite 0·1 grain. This matter was insoluble in acids, but became white. With potash it fused into a colourless glass. Hence I consider it as silica. This experiment shows, that no chromium was present. I was at a loss to account for the precipitate thrown down by the nitrate of mercury. But Mr. Allan having shown me a letter from Ekeberg, in which he mentions, that he had detected muriatic acid in sodalite, it was easy to see that the white precipitate was calomel. The white powder weighed 26 grains, indicating, according to the analysis of Chenevix, about three grains of muriatic acid.

3. The solution, thus freed from muriatic acid, being concentrated by evaporation, gelatinised. It was evaporated nearly to dryness; the dry mass digested in hot water acidulated with nitric acid, and poured upon the filter. The powder retained upon the filter was washed, dried, and heated to redness. It weighed 37·2 grains, and was silica.

4. The liquor which had passed through the filter was supersaturated with carbonate of potash, and the copious white precipitate which fell collected by the filter, and boiled while yet moist in potash-lie. The bulk diminished greatly, and the undissolved portion assumed a black colour, owing to some oxide of mercury with which it was contaminated.

5. The potash-lie being passed through the filter, to free it from the undissolved matter, was mixed with a sufficient quantity of sal-ammoniac. A copious white precipitate fell, which being collected, washed, dried, and heated to redness, weighed 27·7 grains. This powder being digested in sulphuric acid, dissolved, except 0·22 of a grain of silica. Sulphate of potash being added, and the solution set aside, it yielded alum crystals to the very last drop. Hence the 27·48 grains of dissolved powder were alumina.

6. The black residue, which the potash-lie had not taken up, was dissolved in diluted sulphuric acid. The solution being evaporated to dryness, and the residue digested in hot water, a white soft powder remained, which, heated to redness, weighed 3·6 grains, and was sulphate of lime, equivalent to about 2 grains of lime.

7. The liquid from which the sulphate of lime was separated, being exactly neutralized by ammonia, succinate of ammonia was dropped in; a brownish red precipitate fell, which, being heated to redness in a covered crucible, weighed one grain, and was black oxide of iron.

8. The residual liquor being now examined by different reagents, nothing farther could be precipitated from it.

9. The liquid (No. 4.) from which the alumina, lime, and iron had been separated by carbonate of potash, being boiled for some time, let fall a small quantity of yellow-coloured matter. This matter being digested in diluted sulphuric acid, partly dissolved, with effervescence; but a portion remained undissolved, weighing 1 grain. It was insoluble in acids, and with potash melted into a colourless glass. It was therefore silica. The sulphuric acid solution being evaporated to dryness, left a residue, which possessed the properties of sulphate of lime, and which weighed 1·2 grains, equivalent to about 0·7 of a grain of lime.

10. The constituents obtained by the preceding analysis being obviously defective, it remained to examine whether the mineral, according to the conjecture of Bournon, contained an alkali. For this purpose, 100 grains of it, reduced to a fine powder, and mixed with 500 grains of nitrate of barytes, were exposed for an hour to a red heat, in a porcelain crucible. The fused mass was softened with water, and treated with muriatic acid. The whole dissolved, except 25 grains of a white powder, which proved on examination to be silica. The muriatic acid solution was mixed with sulphuric acid, evaporated to dryness; the residue, digested in hot water, and filtered, to separate the sulphate of barytes. The liquid was now mixed with an excess of carbonate of ammonia, boiled for an instant or two, and then filtered, to separate the earth and iron precipitated by the ammonia. The liquid was evaporated to dryness, and the dry mass obtained exposed to a red heat in a silver crucible. The residue was dissolved in water, and exposed in the open air to spontaneous evaporation. The whole gradually shot into regular

 crystals crystals of sulphate of soda. This salt, being exposed to a strong red heat, weighed 50 grains, indicating, according to Berthollet's late analysis, 23·5 grains of pure soda. It deserves to be mentioned, that during this process the silver crucible was acted on, and a small portion of it was afterward found among the sulphate of soda. This portion was separated before the sulphate of soda was weighed.

The preceding analysis gives us the constituents of sodalite as follows:

| | |
|---|---|
| Silica . . . . . | 38·52 |
| Alumina . . . | 27·48 |
| Lime . . . . . | 2·70 |
| Oxide of iron . . . | 1·00 |
| Soda . . . . . | 23·50 |
| Muriatic acid . . | 3·00 |
| Volatile matter . . | 2·10 |
| Loss . . . . . | 1·70 |
| | 100·00 |

Mr. Allan sent a specimen of this mineral to Mr. Ekeberg, who analysed it in the course of last summer. The constituents which he obtained, as he states them in a letter to Mr. Allan, are as follows:

| | |
|---|---|
| Silica . . . . . | 36· |
| Alumina . . . | 32· |
| Soda . . . . . | 25· |
| Muriatic acid . . | 6·75 |
| Oxide of iron . . | 0·25 |
| | 100·00 |

This result does not differ much from mine. The quantity of muriatic acid is much greater than mine. The lime and the volatile matter, which I obtained, escaped his notice altogether. If we were to add them to the alumina, it would make the two analyses almost the same. No mineral has hitherto been found containing nearly so much *soda* as this. Hence the reason of the name by which I have distinguished it.

